# OWL model of clinical trial eligibility criteria compatible with partially-known information

**DOI:** 10.1186/2041-1480-4-17

**Published:** 2013-09-13

**Authors:** Olivier Dameron, Paolo Besana, Oussama Zekri, Annabel Bourdé, Anita Burgun, Marc Cuggia

**Affiliations:** 1, Université de Rennes1, UMR936, F-35000 Rennes, France; 2, INSERM UMR936, F-35000 Rennes, France; 3, Centre Régional de Lutte Contre le Cancer Eugène Marquis, F-35000 Rennes, France

## Abstract

**Background:**

Clinical trials are important for patients, for researchers and for companies. One of the major bottlenecks is patient recruitment. This task requires the matching of a large volume of information about the patient with numerous eligibility criteria, in a logically-complex combination. Moreover, some of the patient’s information necessary to determine the status of the eligibility criteria may not be available at the time of pre-screening.

**Results:**

We showed that the classic approach based on negation as failure over-estimates rejection when confronted with partially-known information about the eligibility criteria because it ignores the distinction between a trial for which patient eligibility should be rejected and trials for which patient eligibility cannot be asserted. We have also shown that 58.64% of the values were unknown in the 286 prostate cancer cases examined during the weekly urology multidisciplinary meetings at Rennes’ university hospital between October 2008 and March 2009.

We propose an OWL design pattern for modeling eligibility criteria based on the open world assumption to address the missing information problem. We validate our model on a fictitious clinical trial and evaluate it on two real clinical trials. Our approach successfully distinguished clinical trials for which the patient is eligible, clinical trials for which we know that the patient is not eligible and clinical trials for which the patient may be eligible provided that further pieces of information (which we can identify) can be obtained.

**Conclusions:**

OWL-based reasoning based on the open world assumption provides an adequate framework for distinguishing those patients who can confidently be rejected from those whose status cannot be determined. The expected benefits are a reduction of the workload of the physicians and a higher efficiency by allowing them to focus on the patients whose eligibility actually require expertise.

## Introduction

Patient recruitment is a major focus in all clinical trials. Adequate enrollment provides a base for projected participant retention, resulting in evaluative patient data. Identification of eligible patients for clinical trials (from the principal investigator’s perspective) or identification of clinical trials in which the patient can be enrolled (from the patient’s perspective) is an essential phase of clinical research and an active area of medical informatics research. The National Cancer Institute has identified several barriers that health care professionals claim in regard to clinical trial participation [1]. Among those barriers, lack of awareness of appropriate clinical trials is frequently mentioned.

Automated tools that help perform a systematic screening either of the potential clinical trials for a patient, or of the potential patients for a clinical trial could overcome this barrier [2]. Efforts have been dedicated to provide a uniform access to heterogeneous data from different sources. The Biomedical Translational Research Information System (BTRIS) is being developed at NIH to consolidate clinical research data [3]. It is intended to simplify data access and analysis of data from active clinical trials and to facilitate reuse of existing data to answer new questions. STRIDE [4] is a platform supporting clinical and translational research consisting of a clinical data warehouse, an application development framework for building research data management applications and a biospecimen data management system. The i2b2 framework integrates medical records and clinical research data [5] and SHRINE [6] handles several sources by providing a federated query tool for clinical data repositories. The ObTiMA system relies on OWL and SWRL to perform semantic mediation between heterogeneous data sources [7]. Lezcano et al. propose an architecture based on OWL to represent patients data from archetypes, and on SWRL rules to perform the reasoning [8]. Several other efforts have been dedicated to the formal representation of clinical trials eligibility criteria to support automated reasoning [9]. Weng et al. performed an extensive literature review [10]. They confirmed that although eligibility criteria are usually written in free text to be human-readble, standard-based computable knowledge representations for eligibility criteria are necessary to clinical and research tasks. They identified five key aspects of eligibility criteria representation, three of which being essential for knowledge-based representation of eligibility criteria: expression language for representing eligibility rules, the encoding of eligibility concepts and patient data modeling. Milian et al. developed a method for automatic formalization of eligibility criteria and comparison of their restrictiveness [11,12]. Their goal is to support the design of eligibility criteria, enable their reuse and provide meaningful suggestions of relaxing them based on previous trials. They processed eligibility criteria from 300 clinical trials, and created a library of structured conditions covering 18% of encountered inclusion and exclusion criteria. Ross et al. conducted a survey of 1,000 criteria randomly selected from ClinicalTrials.gov and found that 80% of them had a significant semantic complexity [13], with 40% involving some temporal reasoning. Tu et al. proposed an approach to convert free text eligibility criteria into the computable ERGO formalism [14]. O’Connor et al. developed a solution based on OWL and SWRL that supports temporal reasoning and bridges the gap between patients specific data and more general eligibility criteria [15].

The ASTEC (Automatic Selection of clinical Trials based on Eligibility Criteria) project aims at automating the search of prostate cancer clinical trials to which patients could be enrolled to [16]. It features syntactic and semantic interoperability between the oncologic electronic medical records and the recruitment decision system using a set of international standards (HL7 and NCIT), and the inference method is based on ERGO [17].

The EHR4CR project aims at facilitating clinical trial design and patient recruitment by developing tools and services that reuse data from heterogeneous electronic health records [18]. The TRANSFoRm project has similar objectives for primary care [19,20].

All these studies on data and criteria representation, integration and reasoning are motivated by the requirement to have the necessary information available at the time of processing the patient’s data, and assume that somehow, that will be the case.

Missing information that is required for deciding whether a criterion is met leads to recruitment being underestimated.

Solutions for circumventing this difficulty consist either in making assumptions about the undecided criteria, or in having a pre-screening phase considering a subset of the criteria for which patient’s data are assumed to be available.

Bayesian belief networks have been used to address the former [21] but require a sensible choice of probability values and may lead to the wrong asumption in particular cases.

The latter leaves most of the decision task to human expertise, which provides little added value (if an expert has to handle the difficult criteria, automatically processing the simple pre-screening ones is only a little weight off his shoulders) and is still susceptible to the problem of missing information for the pre-screening criteria.

We propose an OWL design pattern for modeling clinical trial eligibility criteria. This design pattern is based on the open world assumption for handling missing information. It infers whether a patient is eligible or not for a clinical trial, or if no definitive conclusion can be reached.

## Background

### Modeling eligibility criteria

A clinical trial can be modeled as a pair $<{\left(I_{i}\right)}_{i=0}^{n},{\left(E_{j}\right)}_{j=0}^{m}>$ where ${\left(I_{i}\right)}_{i=0}^{n}$ is the set of the inclusion criteria, and ${\left(E_{j}\right)}_{j=0}^{m}$ is the set of the exclusion criteria. All the eligibility criteria from ${\left(I_{i}\right)}_{i=0}^{n}\cup {\left(E_{j}\right)}_{j=0}^{m}$ are supposed to be independent from one another (at least in the weak sense: the value of criterion *C*_*k*_ cannot be infered from the combined values of other criteria). Each criterion can be modeled as an unary predicate *C*(*p*), where the variable *p* represents all the information available for the patient. *C*(*p*) is true if and only if the criterion is met.

A patient is deemed eligible for a clinical trial if *all* the inclusion criteria and *none* of the exclusion criteria are met.

(1) $$\text{patient eligible}\Leftrightarrow \overset{n}{\underset{i=0}{\wedge }}I_{i}(p)\wedge \neg (\overset{m}{\underset{j=0}{\vee }}E_{j}(p))$$

Before making the final decision on the list of clinical trials for which a patient is eligible for, there are intermediate pre-screening phases where only the main eligibility criteria of each clinical trial are considered. Such pre-screening sessions rely on subsets of ${\left(I_{i}\right)}_{i=0}^{n}$ and ${\left(E_{j}\right)}_{j=0}^{m}$, but the decision process remains the same.

For the sake of clarity, in addition to the general case, we will consider a simple clinical trial with two inclusion criteria *I*_0_ and *I*_1_, and two exclusion criteria *E*_0_ and *E*_1_.

(2) $$\text{patient eligible}\Leftrightarrow I_{0}(p)\wedge I_{1}(p)\wedge \neg (E_{0}(p)\vee E_{1}(p))$$

For example, these criteria could be: 

•*I*_0_: evidence of a prostate adenocarcinoma;

•*I*_1_: absence of metastasis;

•*E*_0_: patient older than 70 years old;

•*E*_1_: evidence of diabetes.

According to equation 2, a patient would be eligible for the clinical trial if and only if he has a prostate adenocarcinoma and has no metastasis and is neither older than 70 years old nor suffers from diabetes.

Because of De Morgan’s laws, equation 1 is equivalent to:

(3) $$\text{patient eligible}\Leftrightarrow (\wedge_{i=0}^{n}I_{i}(p))\wedge (\overset{m}{\underset{j=0}{\wedge }}\neg E_{j}(p))$$

Even though equation 1 and equation 3 are logically equivalent, the latter is often preferred because it is an uniform conjunction of criteria. Note that the negations in front of the exclusion criteria are purely formal, as both inclusion and exclusion criteria can represent an asserted presence (e.g. prostate adenocarcinoma for *I*_0_ or of diabetes for *E*_1_) or an asserted absence (e.g. metastasis for *I*_1_).

For our example:

(4) $$\text{patient eligible}\Leftrightarrow I_{0}(p)\wedge I_{1}(p)\wedge (\neg E_{0}(p))\wedge (\neg E_{1}(p))$$

According to equation 3, a patient would be eligible for the clinical trial if and only if he has a prostate adenocarcinoma and has no metastasis and is not older than 70 years old and does not suffer from diabetes.

### The problem of unknown information

#### Distinction between the patients that we know are not eligible and those that we do not know if they are eligible

When a part of the information necessary for determining if at least one criterion is met is unknown, the conjunction of equation 3 can never be true. This necessarily makes the patient not eligible for the clinical trial, whereas the correct interpretation of the situation is that the patient cannot be proven to be eligible. This is different from proving that the patient is not eligible, and indeed, in reality the patient can sometimes be included by assuming the missing values (cf. next section).

For our fictitious clinical trial, we consider a population of nine patients covering all the combinations of “*True*”, “*False*” or “*Unknown*” for the inclusion criterion *I*_1_ and the exclusion criterion *E*_1_. Table 1 presents the value of equation 4 and correct inclusion decision for the nine combinations. Among the five patients (*p*_2_, *p*_5_, *p*_6_, *p*_7_ and *p*_8_) for which at least a part of the information is unknown, three (*p*_2_, *p*_7_ and *p*_8_) illustrate a conflict between the value of equation 4 and expected inclusion decision. A strict interpretation of equation 4 leads to the exclusion of the eight patients: 

•for three of them (*p*_0_, *p*_3_ and *p*_4_), all the information is available;

•for two of them (*p*_5_ and *p*_6_), some information is unknown, but the available information is sufficient to conclude that the patients are not eligible;

•for the three others (*p*_2_, *p*_7_ and *p*_8_), however, the cause of rejection is either because one of the inclusion criteria cannot be proven (*I*_1_ for *p*_7_ and *p*_8_) or because one of the exclusion criteria cannot be proven to be false (*E*_1_ for *p*_2_ and *p*_8_).

**Table 1:** **Differences between the logical evaluation of the criteria conjunction and the correct inclusion decision when only a portion of the necessary information is known: evaluation of equation**4**and correct inclusion decision for all the possible values of*I***_**1**_** and*E***_**1**_**, with possibly unknown information**

**Patient**	***I***_**0**_	***I***_**1**_	***E***_**0**_	***E***_**1**_	***I***_**0**_**∧*I***_**1**_**∧**	**Decision**
					**¬*E***_**0**_**∧¬*E***_**1**_	
*p*_0_	T	T	F	T	F	Exclude
						(*E*_1_)
*p*_1_	T	T	F	F	T	Include
*p*_2_	T	T	F	?	F	Propose
					cannot	(assume ¬*E*_1_)
					assert ¬*E*_1_	
*p*_3_	T	F	F	T	F	Exclude
						(both ¬*I*_1_ and *E*_1_)
*p*_4_	T	F	F	F	F	Exclude
						(¬*I*_1_)
*p*_5_	T	F	F	?	F	Exclude
						(¬*I*_1_)
*p*_6_	T	?	F	T	F	Exclude
						(*E*_1_)
*p*_7_	T	?	F	F	F	Propose
					cannot	(assume *I*_1_)
					assert *I*_1_	
					F	
*p*_8_	T	?	F	?	cannot	Propose
					assert *I*_1_	
					cannot	(assume both
					assert ¬*E*_1_	*I*_1_ and ¬*E*_1_)

In the case of unknown information, equation 3 alone is not enough to make the distinction between the patients we know are not eligible (the first two categories, so this also includes patients for whom a part of the information is unknown) and those we do not know if they are eligible (the third category). This is a problem because patients from the first two categories should be excluded from the clinical trial, whereas those from the third category should be considered for inclusion.

#### Assuming values for criteria

Currently, the case of each patient diagnosed with cancer is examined in a multidisciplinary meeting (MDM) gathering experts (oncologists, pathologists, surgeons,...). The goal is to determine collectively the best therapeutic strategy for the patient, including consideration of potential inclusion into clinical trials. This preliminary stage is called pre-screening because it takes place before obtaining the patient’s informed consent (i.e., before enrollment). It mainly relies on retrospective data coming from the patient health record. At this point, all the information necessary for determining the status of each inclusion and exclusion criteria may not be available, but the rationale is to focus on the clinical trials for which the patient may be eligible for. It should be noted that the missing items may differ between patients. One solution could be to assume the values of the unknown criteria in order to go back to a situation where inclusion or exclusion could be computed using equation 3.

In this case: 

•inclusion criteria for which the available information is not sufficient to compute the status are considered to be met;

•exclusion criteria for which the available information is not sufficient to compute the status are considered not to be met.

Therefore, in the case where the available information is not sufficient to compute the status of a criterion, a different status is assumed depending on whether the criterion determines inclusion or exclusion.

Referring to our fictitious clinical trial, the lack of information about the absence of metastasis would lead to the assumption that *I*_1_ is true, whereas the lack of information about diabetes would lead to the assumption that *E*_1_ is false.

This situation raises several issues: 

•a different status is assumed depending on whether the criterion determines inclusion or exclusion;

•the assumed status depends on the nature of the criterion (i.e. inclusion or exclusion) and not on its probability;

•one has to remember that the value for at least a criterion has been assumed in order to qualify the inferred eligibility (adamant for *p*_0_ or *p*_1_ vs “under the assumption that...” for *p*_2_, *p*_7_ and *p*_8_);

•this qualification can be difficult to compute (the status of *E*_1_ is unknown for both *p*_2_ and *p*_5_, but *p*_5_ can be confidently excluded whereas *p*_2_ can be included assuming *E*_1_).

### The extent of the missing information problem

To determine the extent of the missing information problem, we analyzed the 286 prostate cancer cases examined during the weekly urology multidisciplinary meetings at Rennes’ university hospital between October 2008 and March 2009. This involved 252 patients: 25 of them were examined during two different MDM, and 5 were examined during three different MDM. Before the MDM, the patient’s data are collected in a form with 65 fields. The form supports the distinction between known and unknown values (e.g. for “antecedent of neoplasm”, the possible answer are “yes”, “no”, “not specified”).

Overall, 11,323 values (60.90%) were not specified. On average, for each case studied in a MDM, 39.6 fields (among 65) had an unknown value.

All of the 286 cases studied had at least some of the 65 fields with an unknown value. Indeed, the case with the most fields filled still missed 22 of them.

59 fields (90.77% of 65) had a missing value in at least one of the 286 cases. The six fields that were systematically filled were: the patient identifier, the MDM date, the patient’s birth date, the patient’s gender, the tumor anatomic site and the primary histological type.

During this period, 4 clinical trials related to prostate cancer running at Rennes Comprehensive Cancer Centre were considered during the MDM. Table 2 presents the composition of the clinical trials fields and their proportion of missing information. It shows that for each clinical trial, all the patients had at least one missing field that prevented formula 3 to be true (regardless of the values of the known fields).

**Table 2:** Importance of unknown information during pre-screening for the four clinical trials of interest: importance of unknown information during pre-screening for the four clinical trials of interest

	**CT1**	**CT2**	**CT3**	**CT4**
Nb inclusion fields	15	19	16	10
Nb exclusion fields	10	9	8	11
Nb common fields	3	0	2	3
Missing values	50.06%	61.72%	56.52%	42.99%
Nb patients with all inclusion fields known	0	0	1	1
Nb patients with all exclusion fields known	4	3	0	1
Nb patients with all fields known	0	0	0	0
Nb eligible patients	30	23	6	2

## Methods

We propose an OWL design pattern for modeling clinical trial eligibility criteria. We then explain how the reasoning unfolds using the fictitious clinical trial from Table 1. We validate our approach by verifying if the inferred outcome corresponds to the expected value from Table 1. We evaluate our approach on two of the four clinical trials related to prostate cancer and the 286 cases mentioned in the previous section. This allows us to quantify the impact of missing information on inclusion rates, as we have seen that in some cases, even partially-known information can lead to certain rejection.

We reused anonymized data from the patients’ medical records and did not conduct any experimental study. The study was approved by Rennes’ Hospital ethics evaluation committee institutional review board under the reference 13-26 (2013).

## Results

### Eligibility criteria design pattern

**Figure 1:**
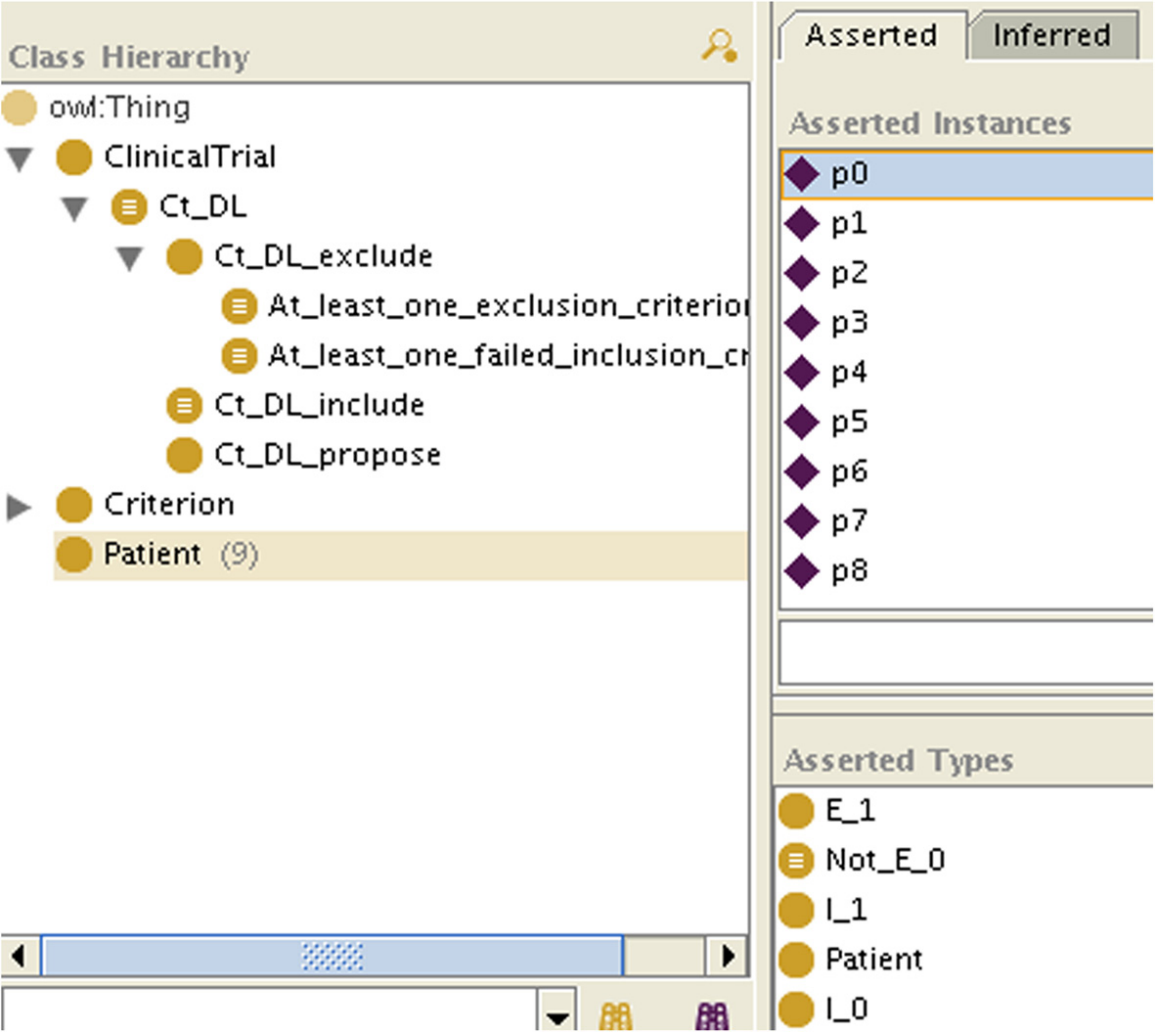
A patient for whom all the information is available.

**Figure 2:**
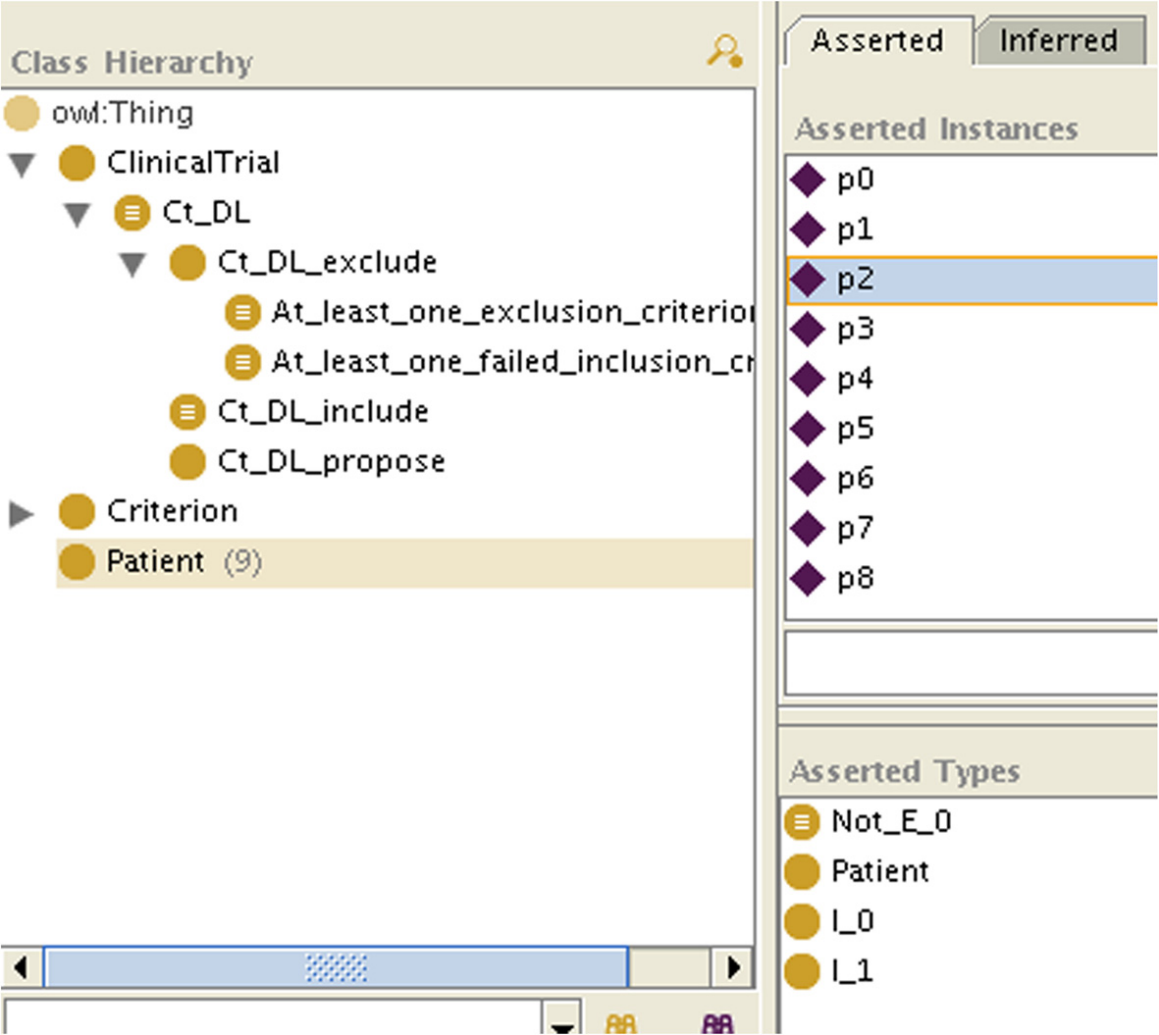
**A patient for whom some information is unknown (here about*E***_**1**_**).**

•for each criterion, create a class C_i (at this point, we do not care if it is an inclusion or an exclusion criteria, or both) and possibly add a necessary and sufficient definition representing the criterion itself (or use SWRL);

•for each criterion, create a class Not_C_i defined as Not_C_i ≡Criterion ⊓¬C_i. This process can be automated;

•for each clinical trial, create a class Ct_k (placeholder);

•for each clinical trial, create a class Ct_k_include as a subclass of Ct_k with a necessary and sufficient definition representing the conjunction of the inclusion criteria and of the exclusion criteria (cf. equation 3) (Ct_k_include$\equiv \overset{n}{\underset{i=0}{⊓}}$I_i$⊓\overset{m}{\underset{j=0}{⊓}}$Not_E_j);

•for each clinical trial, create a class Ct_k_exclude (placeholder) as a subclass of Ct_k;

•for each clinical trial, create a class Ct_k_exclude_at_least_one_exclusion_criterion as a subclass of Ct_k_exclude with a necessary and sufficient definition representing the disjunction of the exclusion criteria (Ct_k_exclude_at_least_one_exclusion_criterion$\equiv \overset{m}{\underset{j=0}{⊔}}$E_j);

•for each clinical trial, create a class Ct_k_exclude_at_least_one_failed_inclusion_criterion as a subclass of Ct_k_exclude with a necessary and sufficient definition representing the disjunction of the negated inclusion criteria (Ct_k_exclude_at_least_one_failed_ incl_criterion$\equiv \overset{n}{\underset{i=0}{⊔}}$Not_I_i);

•represent the patient’s data with instances (Figures 1 and 2). For the sake of simplicity, we will make the patient an instance of as many C_i as we know he matches criteria, and as many Not_C_j classes as we know he does not match criteria, even if this is ontologically questionable (a patient is not an instance of a criterion). How the patient’s data are reconciled with the criteria by making the patient an instance of the criteria is not specified here: it can be manually, or automatically with OWL necessary and sufficient definitions or SWRL rules for the C_i and Not_C_j classes.

### Reasoning

**Figure 3:**
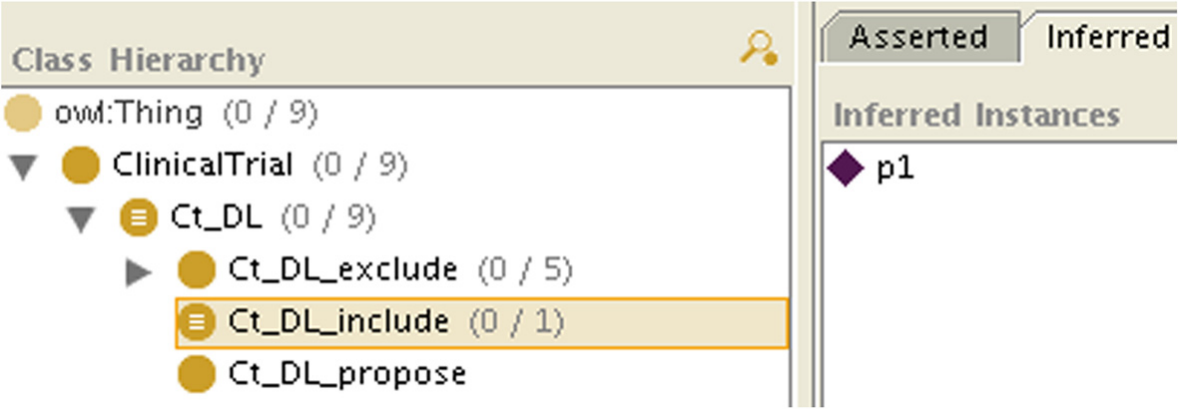
**The class modeling clinical trial inclusion after classification (here patient*p***_**1**_** can be included).**

If all the required information is available, after classification, for each criterion the patient will be an instance of each C_i or Not_C_i, and therefore will also be instantiated as either Ct_k_include (like *p*_1_ in Figure 3), Ct_k_exclude_at_least_one_exclusion_criterion or Ct_k_exclude_at_least_one_failed_inclusion_ criterion (so at least we are doing as well as the other systems).

**Figure 4:**
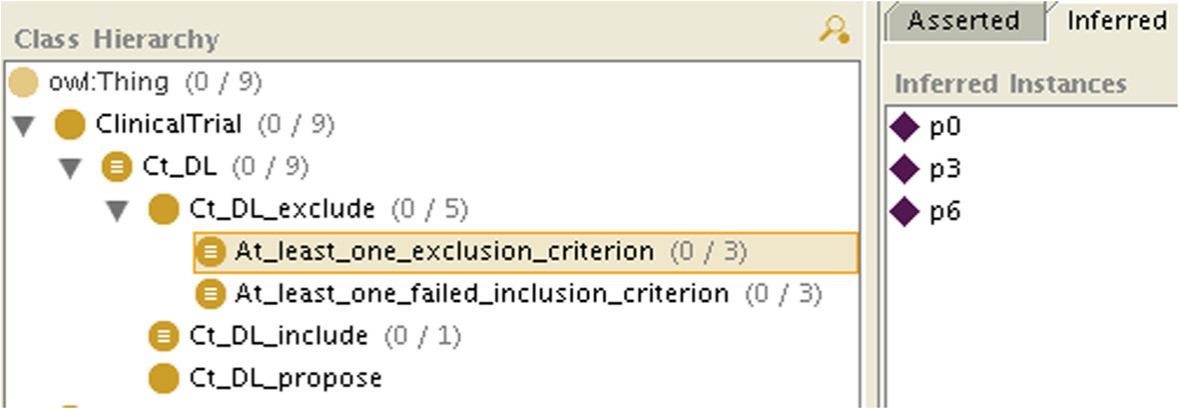
**The class modeling clinical trial exclusion because at least one of the exclusion criteria has been met after classification (here patients*p***_**0**_**,*p***_**3**_** and*p***_**6**_** match the definition).**

**Figure 5:**
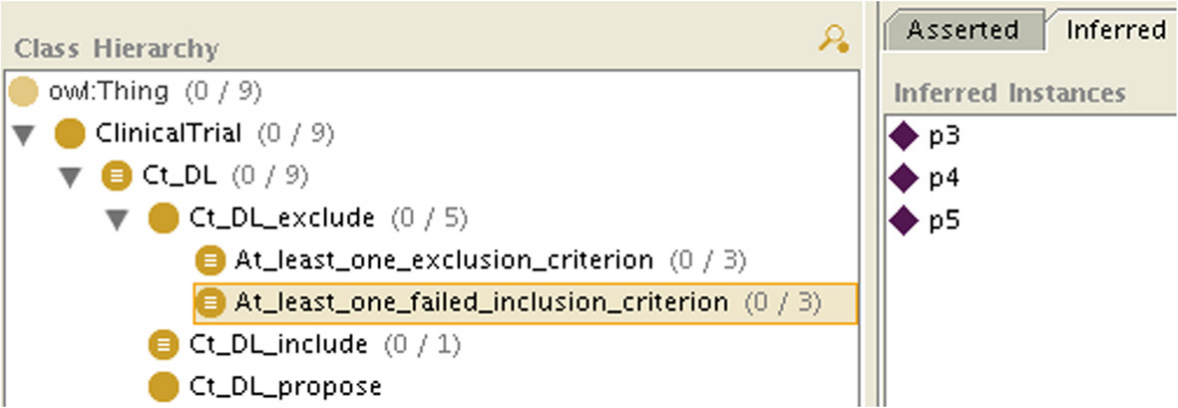
**The class modeling clinical trial exclusion because at least one of the inclusion criteria failed to be met after classification (here patients*p***_**3**_**,*p***_**4**_** and*p***_**5**_** match the definition).**

If not all the information is available, because of the open world assumption, there will be some criteria for which the patient will neither be classified as an instance of C_i nor of Not_C_i (e.g. in Figure 2, *p*_2_ is neither an instance of E_1 nor of Not_E_1), so he will not be classified as an instance of Ct_k_include either. However, the patient may be classified as an instance of Ct_k_exclude_at_least_one_exclusion_criterion or of Ct_k_exclude_at_least_one_failed_inclusion_criterion. As both are subclasses of Ct_k_exclude, we will conclude that the patient is not eligible for the clinical trial. We will even know if it is because he matched an exclusion criterion (like *p*_0_, *p*_3_ and *p*_6_ in Figure 4), because he failed to match an inclusion criterion (like *p*_3_, *p*_4_ and *p*_5_ in Figure 5), or both (like *p*_3_).

If the patient is neither classified as an instance of Ct_k_include nor of Ct_k_exclude (or its subclasses), then we will conclude that the patient can be considered for the clinical trial, assuming the missing information will not prevent it (like *p*_2_, *p*_7_ and *p*_8_, who do not appear in Figures 3, 4 and 5, consistently with Table 1. By retrieving the criteria for which the patient is neither an instance of C_i nor of Not_C_i, we will know which information is missing.

### Validation

We modeled our fictitious clinical trial as well as the nine combinations of values (Additional file 1). All the results were identical to the decision of Table 1.

### Evaluation

We evaluated our model on the first (Additional file 2) and third (Additional file 3) clinical trials.

#### First clinical trial

According to our system, among the 286 cases, 0 were formally eligible, 149 were potentially eligible, and 137 were not eligible. The 30 cases that were identified as eligible by the experts during the multidisciplinary meetings were all among the 149 proposed by our system (precision was 0.20; recall was 1.0).

It should be noted that the *a posteriori* analysis of the 119 cases proposed by our model but not by the MDM revealed that several were not proposed even if they formally met the eligibility criteria because their Gleason score was deemed too low. We added an inclusion criterion requiring patients to have a Gleason score superior or equal to 7. This resulted in 67 cases potentially eligible, among which were 24 of the 30 actually eligible (precision was 0.36; recall was 0.80). The six false negative cases had a Gleason score of 6. Among the 43 false positive, at least 15 were rejected during the MDM because of additional information not available at the time of pre-screening: 8 because new results indicated that they did not have cancer, 3 because too much information was missing and 4 because other elements such as a relatively young age resulted in proposing a surgical treatment instead of the clinical trial.

#### Third clinical trial

According to our system, among the 286 cases, 0 were formally eligible, 34 were potentially eligible, and 252 were not eligible. The 6 cases that were identified as eligible by the experts during the multidisciplinary meetings were all among the 34 proposed by our system (precision was 0.18; recall was 1.0). Among the 28 false positive, 6 cases were rejected during the MDM because of additional information not available at the time of pre-screening, 5 were rejected on the basis of information present in their report but erroneously missing in the database, 15 were rejected because there was no evidence of recurring cancer (not all the cases examined during the MDM of urology have cancer even if most do), and 2 cases were rejected because too much information was missing.

Adding implicit inclusion criteria for performing the same post-processing as the first clinical trial resulted in only 17 potentially eligible cases, among which were 3 of the 6 identified by the experts (precision was 0.18; recall was 0.5). This shows that this strategy is not relevant for this clinical trial.

## Discussion

The observed proportion of missing information is compatible with results from other studies [22]. Köpcke et al. compared the information from 706 patient to 351 eligibility criteria from 15 clinical trials. They reported that the total completeness of EHR data for recruitment purposes was 35%.

The analysis of the first clinical trial demonstrates that missing information would have led to the rejection of all the patients proposed as eligible by the experts during the multidisciplinary meetings. Our approach identified potentially eligible patients (149 for the first clinical trial, and 34 for the third), among which were all the patients deemed eligible by the experts (30 for the first clinical trial, and 6 for the third).

This shows that our system confidently rejects non-eligible cases, which leaves more time to examine the others during the multidisciplinary meetings. Moreover, in the first clinical trial, precision can be significatively improved by adding pragmatic criteria that further discriminate the patients who would not be considered as eligible even if they meet the pre-screening criteria. Note that this second step can be kept separate from the formal determination of eligibility but is useful both for the acceptance of the system by the experts and for maintaining the efficiency of the multidisciplinary meetings.

Missing information can partially be handled even with reasoning based on negation as failure using *ad hoc* conversion between inclusion and exclusion criteria. For example, the inclusion criterion “*absence of ischemic heart disease*” can be converted into the exclusion criterion “*presence of ischemic heart disease*”. The former will probably never be met because a patient’s record only mentions ischemic heart disease when they are present, whereas the latter will (correctly) only exclude those patients having evidence of ischemic heart disease. The problem is that if “*absence of ischemic heart disease*” had been an exclusion criterion, it would likewise have been converted into the inclusion criterion “*presence of ischemic heart disease*” and the system would have (incorrectly, at least during pre-screening) rejected patients whose record does not mention the presence nor the absence of ischemic heart disease. Moreover, a criterion can be an inclusion criterion for a clinical trial and an exclusion criterion for another trial, so this strategy is not a general solution to the problem of missing information.

Reasoning about the conjunction of the eligibility criteria should be handled by OWL, which supports the open world assumption, rather than by related technologies such as SWRL which do not. It would be possible to write a SWRL rule that represents the conjunction of criteria (cf. formula 3). However, it is impossible to distinguish situations where we know that one criterion is not met from those where we cannot determine if it is met, because in both cases the rule will not fire.

Applying our criteria modeling design pattern to real clinical trials and real patients’ data was a manual process. The reasoning part of our contribution focused on combining the status of the eligibility criteria when some of then can not be determined, not on determining the statuses themselves. However, both points are of importance. Our design pattern consisted in modeling each criterion by two classes representing the certain presence and the certain absence of the criterion for a patient. As we have seen in this article, this first modeling part was easy, can be automated, and addressed the problem of missing information as one of the causes of patient recruitment underestimation. When evaluating our system on real clinical trials and real patients’ data, we had to determine for each patient whether each criterion was met. This required both the occasional decomposition of complex criteria into logical combinations of simpler conditions, and the binding with the patients’ data representation in the local EHR. The first step is generic and rather straightforward. It only has to be done once, and can be reused shared between hospitals or reused if a criterion appears in several clinical trials. The second step is clearly dependent on the local representation of patients’ data, and was more difficult and labor-intensive. It also required to write the functions that process the data, which took a couple of days for each clinical trial (a portion of the code written for the first CT could be reused for the second one).

The standardization of data elements would provide a significant help to the challenge of connecting the patients’ data with the eligibility criteria. The main standard organizations (HL7,OpenEHR/EN213606 for clinical care) and CDISC [23] (for clinical research domain) define their own semantic interoperability framework to structure and encode data elements with reference terminologies. Moreover recent initiatives have been carried out to fill the gap between clinical data sources coming from EHRs and Clinical Data Management Systems (CDMS) including Recruitment Support Systems. For instance, the Joint Initiative Council was formed as a partnership between HL7, CDISC, ISO TC 215, IHTSDO, and CEN TC 251 with the stated goal of increasing collaboration between standards organizations based on the recognition of a common goal of computable semantic interoperability. Clinical Data Acquisition Standards Harmonization (CDASH) is an initiative that specifies the unambiguous semantics of a number of common data elements that are deemed “common” to all trials. As such, CDASH represents a significant first-step in achieving cross-trial semantic interoperability. BRIDG [24] (Biomedical Research Integrated Domain Group) model which, on one side, contains representations of clinical research data with underlying mappings to the HL7 RIM and, on the other side, covers a superset of the scope defined by CDASH. Currently, several projects around the world are currently using these standards such as REUSE [25], EHR4CR [18,26], TRANSFORM [19,20] or CaBIG [27].

The use of RDF-based (Resource Description Framework) Semantic Web formats (hopefully standardized) data elements and eligibility criteria would also make their integration easier. RDF proved to be a key elements for data integration in more general contexts. Associated querying and reasoning techniques based on SPARQL (SPARQL Protocol and RDF Query Language) and SPIN (SPARQL Inference Notation) for determining the status of eligibility criteria would have the advantage of having the rules represented in the same language as the schema and data to which those rules are attached, as well as having sustainable computation performances. On the other hand, these strategies usually rely on closed-world reasoning. Future work should focus on studying the benefits of such an approach and on determining how well it can address the problem of missing information.

Potential applications of our approach are not limited to clinical trials [21]. They cover all clinical decision situations where some information may be missing. We are currently adapting this approach for the determination of pacemaker alerts severity [28]. Electronic health records and clinical reports have been shown to exhibit large amounts of redundant information [29,30], but Pakhomov et al. observed a discordance between patient-reported symptoms and their (lack of) documentation in the electronic medical records [31]. They noted that this has important implications for research studies that rely on symptom information for patient identification and may have clinical implications that must be evaluated for potential impact on quality of care, patient safety, and outcomes.

## Conclusions

We have shown that ignoring the missing information problem for automatic determination of clinical trial eligibility led to over-estimate rejection. Systems based on negation as failure infer that the patient is not eligible if it cannot be proved that he is eligible, whereas the situations where it cannot be determined that the patient is eligible nor that he is not eligible should be identified and treated separately. A retrospective analysis of 252 patients with prostate cancer showed that for the four clinical trials of interest, all the patients had at least one missing value that resulted in their rejection whereas 62 of them were actually eligible for at least one of the clinical trials.

We proposed a modeling strategy of eligibility criteria in OWL that leveraged the open world assumption to address the missing information problem. Our approach was able to distinguish a clinical trial for which the patient is eligible, a clinical trial for which we know that the patient is not eligible and a clinical trial for which the patient may be eligible provided that further pieces of information (which we can identify) can be obtained.

By confidently rejecting some of the non-eligible cases, our approach leaves more time to examine those requiring medical expertise during the multidisciplinary meetings.

## Competing interests

The authors declare that they have no competing interests.

## Authors’ contributions

OD designed the study, implemented the reasoning mechanism and drafted the manuscript. PB and AB implemented the patient’s data repository. OZ collected the patient’s data and provided medical expertise. AB and MC revised the manuscript. All authors read and approved the final manuscript.

## Supplementary Material

Additional file 1 **OWL files for the validation set.** The file clinicalTrial-validation.tgz is a zipped tarball containing a readme.txt and the OWL files modeling the criteria and the patients’ data from the validation set. Click here for file

Additional file 2 **OWL files for the first clinical trial of evalution set.** The file clinicalTrial-getug14.tgz is a zipped tarball containing a readme.txt and the OWL files modeling the criteria and the patients’ data for the first clinical trial from the evaluation set. Click here for file

Additional file 3 **OWL files for the third clinical trial of evalution set.** The file clinicalTrial-getug16.tgz is a zipped tarball containing a readme.txt and the OWL files modeling the criteria and the patients’ data for the first clinical trial from the evaluation set. Click here for file
